# Foliar-applied silicate potassium modulates growth, phytochemical, and physiological traits in *Cichorium intybus* L. under salinity stress

**DOI:** 10.1186/s12870-024-05015-6

**Published:** 2024-04-16

**Authors:** Hamid Mohammadi, Soraya Abdollahi-Bastam, Ahmad Aghaee, Mansour Ghorbanpour

**Affiliations:** 1 Faculty of Agriculture, Azarbaijan Shahid Madani University, Tabriz, Iran; 2 Department of Biology, Faculty of Science, University of Maragheh, Maragheh, Iran; 3 Department of Medicinal Plants, Faculty of Agriculture and Natural Resources, Arak University, Arak, 38156-8-8349 Iran

**Keywords:** *Cichorium intybus* L., K_2_O_3_Si, Stress physiology, Salinity, Secondary compounds

## Abstract

One of the major problems endangering plant growth and productivity worldwide is salt stress. This study aimed to assess the effects of potassium silicate (K_2_O_3_Si) on the physical, biochemical, and morphological characteristics of chicory (*Cichorium intybus* L.) under various levels of salinity stress. The plants were treated with K_2_O_3_Si at concentrations of 0, 1, 2, and 3 mM and cultivated under different salt stress conditions (0, 80, 160, and 240 mM NaCl). The findings revealed that salt stress led to decreased root and shoot dry weights, Fv/Fm ratio, chlorophyll a, b, and total chlorophyll, as well as inulin contents. However, foliar exposure to K_2_O_3_Si at all salinity levels resulted in improvements in the measured traits. As salinity levels increased, there was a corresponding increase in the accumulation of sodium ions (Na^+^) and a sharp reduction in potassium ions (K +) in the shoot. Nonetheless, treatment with K_2_O_3_Si caused a decrease in Na + accumulation and an improvement in K^+^ content under all salinity levels. Carotenoid content increased under 80 mM salinity stress, but decreased with higher salinity levels. Application of K_2_O_3_Si at all levels resulted in increased carotenoid content under salinity stress conditions. The content of MDA increased significantly with increasing salinity stress, particularly at 240 mM. However, foliar spraying with K_2_O_3_Si significantly decreased MDA content at all salinity levels. Salinity stress up to 160 mM increased the total phenol, flavonoid, and anthocyanin contents, while 240 mM NaCl decreased the biosynthesis of phytochemicals. Additionally, the use of K_2_O_3_Si increased the content of total phenol, flavonoid, and anthocyanin at all salt levels. Foliar application of K_2_O_3_Si increased the tolerance of chicory plants to salinity stress by reducing MDA and increasing phenolic compounds and potassium content. These results suggest that exogenous K_2_O_3_Si can be a practical strategy to improve the growth and yield of chicory plants exposed to saline environments.

**Supplementary Information:**

The online version contains supplementary material available at 10.1186/s12870-024-05015-6.

## Introduction

The use of medicinal compounds derived from plants has a long history. Even today, the side effects caused by chemical drugs and the numerous inadequacies of modern medicine in the treatment of some diseases have once again increased the attraction towards the cultivation and production of medicinal plants. According to the WHO (World Health Organization) estimate, more than 80% of the world's population still utilizes herbal medicines to treat diseases. In addition, considering that medicinal plants have few side effects, there is a great demand for them all over the world as natural flavors and aromas apart from medical drugs [1].

Chicory (*Cichorium intybus* L.) belongs to the family of Asteraceae. This plant comes in two cultivated species and four to six wild species. Its cultivated type can grow up to two meters, whereas its wild type can grow up to one meter. A herb called chicory has blue or purple blossoms. Chicory originated in the Old World and eventually spread to the Americas as a roadside plant. Its main origin is Central Europe, North Africa, and Western and Central Asia. It also has a wide distribution in different regions of Iran, particularly Euclid city, Azerbaijan, Fesaroud neighborhood, and mountainous areas of Khorasan. It needs a cool, sunny, or slightly shaded climate and cannot tolerate the extreme heat of summer [2]. Chicory is completely cold, and it strengthens the liver relieves heat and thirst, heats and strengthens the kidneys and blood pressure and bile, and cleanses the urinary tract and kidneys. Chicory leaf juice is the best remedy for jaundice, kidneys, and liver, and its decoction is a cure for old fevers, and a stomachic tonic along with sugar flower cures persistent minor fevers. Chicory is very useful for regulating blood pressure [3].

Food security is a critical need for all societies. With the increasing population and the need for more food, coupled with the lack of suitable soils for agriculture, modern societies are facing a serious challenge. World agriculture is tasked with producing 70% more food crops to feed the projected 2.3 billion additional people by 2050 [4]. Salinity, a widespread soil degradation process, hinders the increase in food production to meet rising demand. Salts progressively accumulate in the soil, affecting the entire world. Water-soluble salts significantly impact crop and soil productivity. Reports indicate that over 45 million hectares of irrigated land (20% of the world's total arable land) are affected by salinity stress, and every year 1.5 million hectares of arable land become unusable due to high salinity. It is expected that the increase in salinity will result in the loss of up to 50% of arable land by the mid-twenty-first century [5].

Silicon is the second most abundant component in the Earth’s crust, constituting 27.6% of its composition. While not considered an essential element for plants [6], it can impact several crucial metabolic pathways. For instance, silicon can influence plant water relations, cell wall flexibility, and membrane stability [7].

The negative impact of abiotic stresses such as drought, flooding, salinity, extreme temperatures, toxins, and nutritional deficiencies on crop growth and production poses a threat to global food security [8]. Among these stressors, salinity has the most significant impact on agricultural lands worldwide due to poor quality irrigation water, inadequate drainage, coastal lands with salty water, and salt accumulation in dry areas. Approximately 21% of arable land globally is affected by salinity, making it a major issue in arid and semi-arid regions. The adverse effects of climate change on crops are well recognized, with rising temperatures disrupting weather patterns and leading to regular occurrences of flooding, drought, and salinity. Furthermore, the melting of ice caps and natural glaciers is expected to increase sea levels, potentially exacerbating soil salinity and seriously impacting crop production. Salinity inhibits plant growth in various ways: by increasing osmotic stress, making water absorption difficult, and causing ionic toxicity due to the impact of sodium ions on cellular function, leading to reduced nutrient absorption, photosynthesis, enzyme activity, and metabolism [9]. The initial phase of salinity stress is characterized by the impact of external salt on the root zone, hindering water absorption, cell contraction, root and leaf growth, damage to cells in wet leaves, and reduction of new leaves. In the later phase, salt stress is due to the toxic impact of internal salt. Salinity stress directly affects photosynthesis and causes oxidative stress by reducing the availability of carbon dioxide through limitations in its diffusion via stomata and interleaf transfer. Leaf fall occurs after salinity stress, as nutrients in leaves are transferred to other growing parts [10]. In addition, plants employ various mechanisms to cope with salt stress, such as controlling the absorption and transfer of ions to aerial organs, the accumulation and selective release of ions, precise ion replacement in cells, the synthesis of compatible solutes, and changes in membrane structure. Cells use antioxidant enzymes and plant hormone production to mitigate the adverse effects of salt stress. The response of plants to salinity stress typically occurs in two phases: an initial decrease in ion-independent growth, which causes stomata to close and inhibits cell expansion, and a subsequent phase related to the creation of cytotoxic ion levels, slowing down metabolism and causing premature aging and cell death. Several molecular and physiological mechanisms, such as osmotic tolerance, ionic tolerance, and tissue tolerance, are involved in regulating these responses [5].

One way to reduce the harmful effects of salinity stress is to employ mineral nutrition methods, such as using silicon. In higher plants, silicon typically enhances the physical strength of organs by permeating the stem and leaves, improving physiological and metabolic processes, gas exchanges, and fortifying the antioxidant system. This ultimately boosts the plant's efficiency in coping with various environmental stresses [11].

The chicory medicinal plant is an important species of the Asteraceae family. The leaves and vegetative parts of the chicory plant contain biologically active substances, including essential oils such as camphor, cumin, gamma-terpinene, and cuminal [3]. Given the valuable medicinal compounds present in chicory and its wide use in food and pharmaceuticals, further research on this plant is necessary. Salinity stress is a significant environmental factor that limits plant production worldwide [12]. Studies have shown that in sensitive plant species, salinity causes water loss and ion toxicity, leading to nutrient deficiency, reduced growth, and even plant death. Plants have developed mechanisms to regulate salt accumulation in different organs to cope with salinity. Tolerant plant species are better able to use protective mechanisms against stress conditions compared to sensitive plants. This may involve the distribution of toxic ions among tissues or within cells, or the accumulation of osmolytes that help maintain photosynthetic activity. In addition, the induction of antioxidant systems can be an effective aid in salinity protection [13].

Several studies have demonstrated the positive impact of silicon on plant growth and performance. For example, research on cherry tomato salinity resistance found that silicon had a beneficial effect by protecting photosynthetic activity against the harmful effects of salinity [14]. Another study on zucchini in hydroponic cultivation showed that 1 mM silicon increased vegetative growth, fruit yield, and photosynthesis, while reducing the detrimental effects of salinity [15]. Given the wide application of chicory medicinal plant in Iran and the significant effects of silicon on reducing the adverse impacts of salinity stress, it is important to conduct further research. An experiment was designed to investigate the physiological and phytochemical reactions of chicory medicinal plant to different concentrations of silicon under salinity stress, assess the impact of salinity stress on the growth and performance of chicory medicinal plant, and examine the effects of different concentrations of silicate on chicory’s secondary metabolites.

## Materials and methods

### Plant materials, experimental site and setup

In order to examine the effect of two significant factors, potassium silicate and salinity stress, on dry matter, and the physiological and phytochemical characteristics of the chicory medicinal plant, a greenhouse experiment was performed in the greenhouse of Azarbaijan Shahid Madani University, Tabriz, Iran. The current research was implemented as a factorial design in the form of randomized complete blocks with three replications. The studied treatments included salinity stress (at four levels: control, 80, 160, and 240 mM sodium chloride) and different concentrations of potassium silicate (0, 1, 2, and 3 mM). Ten intact and uniform Chicory seeds were scattered in each 4 L pot on 21 April 2022, and it appeared seedlings were thinned to 6 plants per pot. These pots were reserved in a controlled environment with a relative humidity of 52%–62%, minimum and maximum temperatures of 14 °C and 28 °C, respectively, and a day length of 14 h (using incandescent and fluorescent lamps). Salt stress and potassium silicate treatments were applied after 4 weeks of seed cultivation. Irrigation of pots with varying electrical conductivity (EC) was used to apply salinity treatments, which were produced with sodium chloride (NaCl). A portable EC-meter was used to measure the EC of irrigation water (EC_iw_). The plants were slowly subjected to saline treatment to avoid unexpected stress. The EC of the drainage water of the pots was monitored during each irrigation to maintain the EC of the potting soil and ensure the right application of salinity treatments. Distilled water was used to create the potassium silicate solutions. In the early hours of a sunny day, potassium silicate solutions were sprayed on plant shoots. The 135-day-old chicory plants were harvested completely, with the shoot and root being separated. The samples were all kept in a ventilated oven for 48 hs at 70 ± 2 °C; the dry weights of shoots and roots were then measured with a digital scale with an accuracy of 1 mg. In order to calculate the sodium (Na^+^) and potassium (K^+^) ion concentrations in chicory plants, the dried samples were all ground and turned to ashes in a furnace at 600 °C for 4 h. Then, 10 mL hydrochloric acid (2 N) was added to the ashes of each sample and the mixtures were heated at 90 °C to eliminate hydrochloric acid. The digested ash was dissolved in 100 mL distilled water and filtered. The Na^+^ and K^+^ concentrations were determined by a flame photometer. The Na^+^ and K^+^ contents were calculated using the standard curve.

### Measurements of chlorophyll fluorescence

By a PAM-2000 portable fluorometer (Walz, Effeltrich, Germany) linked to a notebook computer, in vivo Chl. Fluorescence was calculated in completely expanded connected flag leaves in the greenhouse for control, and water-stressed plants were pre-darkened for 1 h. In dark-adapted leaves, the ratio between variable and maximal fluorescence (Fv/Fm) was obtained. The variable's ratio to maximum fluorescence (Fv/Fm) obtained from the measurement was utilized as a measure of the maximum photochemical effectiveness of photosystem II (PS II).

### Plastid pigment measurements

In a mortar with 20mL of distilled acetone, fresh plant materials (0.1 g each) were all ground. The extract was then centrifuged for 10 min at 8000 g. The clear supernatant was made up to 10mL using 80% acetone. At 470 (carotenoids)), 645 (chlorophyll α), and 663 (chlorophyll b) nm, the extract absorbance was read [16].

### The leaf MDA content assessment

Malondialdehyde (MDA) content was measured by the thiobarbituric acid (TBA) reaction [17]. Frozen samples were weighed (0.1 g) and placed in a mortar, then crushed and homogenized with liquid nitrogen. The samples were transferred to tubes, and 1.0% trichloroacetic acid (TCA) (1.5 mL) was added to each tube. The tubes were centrifuged at 12,000 rpm for 10 min at 4 °C. Then, 0.5 mL of the supernatant was taken, and 1.0 mL of 20% TCA containing 0.5% thiobarbituric acid was added. The mixture was incubated in a water bath at 95 °C for 30 min. After that, the mixture was immediately placed in an ice bath to cool down and centrifuged at 10,000 rpm for 10 min. The absorbance of the supernatant was measured at 532 nm, and the non-specific absorption at 600 nm was subtracted. The malondialdehyde concentration was calculated using a correction coefficient of 155 mM^−1^ cm^−1^ and expressed in nmol per gram of fresh weight.

### Total phenolic content

Total phenolic content was determined by the Folin-Ciocalteu method [18]. In brief, 200 mL of crude extract (10 mg/mL) were mixed carefully with 0.5 mL of Folin–Ciocalteu reagent for 3 min, followed by 2 mL of sodium carbonate (20%, w/v), and then the absorbance was measured at 750 nm using a UV–Vis spectrophotometer. The total phenolic content was expressed as milligrams of gallic acid equivalent per gram of dried leaf weight.

### Total flavonoid content determination

Aluminum chloride colorimetry was used to determine flavonoid content [19]. Briefly, 50 µL of extract (1 mg/mL) was mixed with 10 µL of 10% aluminum chloride solution and 10 µL of 1 M sodium acetate. Finally, the total flavonoid content was well-defined as the quercetin equivalent per gram of dried leaf weight (mg QE g^−1^ DW extract) after 15-min incubation at 415 nm.

### Anthocyanin content

Sutharut and Sudarat's technique was utilized to determine anthocyanin content [20]. The total anthocyanin content was determined by the pH-differential method.

### Inulin extraction

The inulin percentage in the root was examined by the methods of Lingyun et al. [21]. In brief, total carbohydrate was determined by the phenol–sulphuric acid method. Reducing sugar was determined by the dinitrosalicylic acid method. The inulin content was measured with the difference between total carbohydrates and reducing sugars.

### Statistical analysis

The statistical analyses were analysis of variance (ANOVA) and mean comparison through Duncan’s multiple range test, done with SAS software V 9.1.

## Results and discussion

### Root and shoot dry weight

Based on the results, the shoot, root dry weight, and root-to-shoot ratio were influenced by the interaction effects of salinity stress and the external application of potassium silicate (Additional file 1). The mean comparison of data showed that spraying with potassium silicate at concentrations of 2 and 3 mM in all four stress levels led to an increase in root and shoot dry weight (Table 1). Salt stress improved the root-to-shoot ratio with potassium silicate application. When salinity increased from 0 to 240 mM NaCl, the root-to-shoot ratio augmented (Table 1), representing that the negotiation of root growth and root-to-shoot ratio might deliberate better tolerance to salt stress. Studies have shown that potassium silicate leads to extensive changes in root growth by increasing the root volume and weight and ultimately increases the dry weight of the root and the absorbing surface of elements [22]. It is also known that silicon leads to a rise in the dry weight of the whole plant through the improvement of the cell wall structure and the availability of elements involved in growth [23]. Potassium silicate is a plant biostimulant rich in potassium and silicon with a very high solubility percentage [24]. Usually, this substance is used in agricultural products by providing small amounts of potassium as a modifier that improves the quality and performance of these products [23]. In addition, potassium silicate increases vegetative growth and yield components, as well as the concentration of mineral nutrients, including phosphorus, nitrogen, and potassium. This substance also impacts physiological functions, e.g., sugar and starch production, cell division, protein synthesis, growth, and fruiting [25]. based on studies, potassium silicate (K_2_SiO_3_) maintains the plasma membrane function through an increase in enzymatic antioxidant activity throughout salinity stress [26]. Based on many reports, this material diminishes environmental stress [23, 25]. The increase in shoot or plant root growth with the help of Si during salt stress has been indicated in numerous plant species, including cucumber, and tomato [27]. Roots have a very important role in plant growth. They are the first tissue to feel salt stress. According to reports, Si regulates plant root growth and structure under salinity stress conditions [28]. This element in cucumber increases the root-to-shoot ratio in plants under salt stress and probably improves the water balance in the plant by improving the hydraulic conductivity of the root [29]. Si may promote root growth by enhancing Casparian strip creation and stimulating lignin and suberin biosynthesis or increasing cell wall tension in the growth zone [30].

**Table 1:** Effect of different salinity stress with foliar silicate potassium application on some studied traits of *Cichorium intybus* L. plants

Salinity stress (mM)	Silicate potassium	Shoot Dry Weight (g)	Root Dry weight (g)	Root-to-shoot ratio	Fv/Fm	Na^+^ content (mg/g DW)	K^+^ content (mg/g DW)	K/Na
0	Control	10.97^c^ ± 0.54	2.85^d^ ± 0.16	25.95^h^ ± 0.28	0.77^a^ ± 0.01	0.15^j^ ± 0.01	16.62^cde^ ± 0.95	113.81^d^ ± 1.02
	0.7	11.17^c^ ± 0.61	2.90^d^ ± 0.17	25.93^h^ ± 0.12	0.78^a^ ± 0.02	0.14^j^ ± 0.02	16.86^bc^ ± 0.92	119.72^c^ ± 1.27
	1.4	12.08^b^ ± 0.66	3.26^b^ ± 0.18	27.02^g^ ± 0.19	0.79^a^ ± 0.01	0.14^j^ ± 0.01	17.38^ab^ ± 0.94	123.44^b^ ± 1.32
	2.1	15.18^a^ ± 0.83	3.42^a^ ± 0.19	22.52^j^ ± 0.21	0.79^a^ ± 0.01	0.13^j^ ± 0.01	17.68^a^ ± 0.96	134.49^a^ ± 1.43
80	Control	6.70^g^ ± 0.38	2.34^g^ ± 0.14	34.93^b^ ± 0.13	0.69^c^ ± 0.02	0.92^h^ ± 0.05	12.76^j^ ± 0.77	13.84^h^ ± 0.54
	0.7	9.38^d^ ± 0.54	2.54^f^ ± 0.15	27.06^g^ ± 0.10	0.74^b^ ± 0.02	0.92^h^ ± 0.04	14.61^hi^ ± 0.89	15.92^g^ ± 0.29
	1.4	10.52^c^ ± 0.60	2.59^ef^ ± 0.16	24.64^i^ ± 0.19	0.74^b^ ± 0.01	0.81^i^ ± 0.04	15.96^ef^ ± 0.97	19.60^f^ ± 0.36
	2.1	12.21^b^ ± 0.70	3.04^c^ ± 0.17	24.89^i^ ± 0.14	0.74^b^ ± 0.01	0.75^i^ ± 0.03	16.68^cd^ ± 0.96	22.26^e^ ± 0.48
160	Control	4.48^h^ ± 0.26	1.57^k^ ± 0.1	34.96^b^ ± 0.13	0.59^e^ ± 0.01	1.67^b^ ± 0.1	10.81^k^ ± 0.66	6.49^k^ ± 0.1
	0.7	6.58^g^ ± 0.38	2.11^h^ ± 0.13	31.99^d^ ± 0.12	0.69^c^ ± 0.02	1.31^d^ ± 0.08	14.41^i^ ± 0.88	10.97^i^ ± 0.31
	1.4	7.36^ef^ ± 0.43	2.40^g^ ± 0.15	32.64^c^ ± 0.15	0.70^c^ ± 0.03	1.11^f^ ± 0.07	15.33^fg^ ± 0.94	13.85^h^ ± 0.28
	2.1	7.73^e^ ± 0.45	2.68^e^ ± 0.17	34.68^b^ ± 0.23	0.71^c^ ± 0.01	1.01^g^ ± 0.06	16.10^de^ ± 0.99	15.97^j^ ± 0.31
240	Control	2.44^i^ ± 0.08	0.76^i^ ± 0.03	30.98^e^ ± 0.33	0.52^f^ ± 0.01	1.95^a^ ± 0.09	8.28^l^ ± 0.36	4.24^l^ ± 0.07
	0.7	4.94^h^ ± 0.16	1.79^j^ ± 0.7	36.11^a^ ± 0.39	0.60^de^ ± 0.01	1.59^c^ ± 0.07	13.30^j^ ± 0.49	8.36^j^ ± 0.08
	1.4	6.78^fg^ ± 0.22	1.92^i^ ± 0.08	28.35^f^ ± 0.31	0.61^de^ ± 0.02	1.30^d^ ± 0.06	14.03^i^ ± 0.52	10.76^i^ ± 0.09
	2.1	6.62^g^ ± 0.21	2.04^hi^ ± 0.09	30.85^e^ ± 0.33	0.62^d^ ± 0.01	1.19^e^ ± 0.05	15.25^gh^ ± 0.56	12.79^h^ ± 0.08

^a,b,c,d,e,f,g,h,i,j,k,l^Means followed by the same letter(s) in each column are not significantly different based on Duncan’s Multiple Range Test (*n* = 3)

### Sodium and potassium contents

The results showed that the interaction effect of salinity stress and spraying with potassium silicate has a momentous impact on the sodium and potassium content of the chicory plant at the probability level of 1% (Additional file 1). The mean comparison of data showed that the increase in salinity level leads to an increase in sodium ion accumulation and a sharp decrease in potassium ions in the shoot part, but the use of potassium silicate, especially in a concentration of 3 mM, led to a decrease in sodium ion accumulation and an improvement in potassium ion content in all salinity levels (Table 1). Studies show that salinity stress through the accumulation of toxic ions causes an imbalance in the absorption of nutrients and the leakage of ions from the membrane [31]. Also, studies indicate that silicon leads to a reduction in the damaging impacts of salinity stress, an improvement in the plant's antioxidant status, and a reduction in ionic toxicity due to less sodium absorption or the improvement in the activity of ATPase proton pumps in the cell membrane and related to potassium ions [32]. Silicon may reduce the adverse impacts of oxidative stress, especially in abiotic stress conditions, through the regulation of reactive oxygen species (ROS) in the antioxidant system [33]. The alternative function of silicon elements is to increase tolerance to stress and improve physiological regulation, including increasing the efficiency of stomata [34]. Potassium (K), among the significant and consumed components in plants, stimulates root length and vegetative growth and regulates osmotic pressure. In addition, this element controls several metabolic activities, including protein production, photosynthesis, water status, pore water transport, and carbohydrate synthesis [26]. After all, it is actively involved in several functions, like activating enzymes and absorbing harmful ions such as Na^+^. Thus, this element may be utilized to minimize the adverse impacts of salinity stress in plants [25]. Much research on the mechanisms through which Si reduces salinity stress in a plant typically focuses on reducing Na^+^ in roots or shoots. For instance, adding Si to the atmosphere under salinity stress conditions can provide the possibility of uniform distribution of Na^+^ and K^+^ in the whole system by significantly reducing the Na^+^ and Cl^−^ levels in the root system. This is among the significant mechanisms through which Si reduces salinity stress [35].

### K/Na ratio

The results showed that salinity stress and the application of potassium silicate simultaneously have a significant effect on the ratio of potassium to sodium (Additional file 1). Salinity stress at all levels resulted in a sharp decrease in the K/Na ratio, but foliar application of potassium silicate improved this ratio (Table 1). Studies show that increasing salinity levels decreases potassium ion content and increases sodium ion content [36]. An enhancement in sodium and chloride content in sodium chloride (NaCl) treatment resulted in a substantial decrease in potassium content in plants. Nevertheless, the K_2_SiO_3_ application reduced Na^+^ accumulation in leaves. The toxicity degree of Na^+^ and Cl^−^ in a metabolic process is due to their competition with K^+^ to binding sites, eventually disrupting the activity of enzymes and vital cell functions. As a result, plants grown in saline soils can suffer from problems caused by low potassium concentration in addition to the damage caused by sodium toxicity [5]. In the present research, the plants sprayed with K_2_SiO_3_ had more potassium than the control sample due to significantly decreased Na^+^ and improved maintenance of K^+^ concentration in the leaves under salinity stress conditions. In addition, this may lead to higher salt tolerance in the plant.

### Fv/Fm

The interaction effect of salinity stress and the usage of potassium silicate positively affected the Fv/Fm ratio (Additional file 1). Salinity stress, especially the level of 240 mM, caused a sharp reduction in the Fv/Fm ratio, and the use of potassium silicate significantly improved the Fv/Fm ratio at all salinity levels (Table 1). Studies show that the Fv/Fm ratio specifies the quantum efficiency of PSII in converting absorbed light into chemical energy, and the reduction of this ratio is caused by salinity stress damage to PSII which ultimately leads to a decrease in photosynthesis under salinity stress conditions [37]. The decrease in the intensity of photosynthesis and the reduction in the Fv/Fm ratio may be associated with the decrease in the relative water content of the leaf, the increase in membrane permeability, the closing of the stomata, and the increase in the accumulation of ions [38]. Foliar spraying with potassium silicate also leads to the improvement of Fv/Fm and photosynthesis through improving the relative moisture content of the leaves, increasing the antioxidant system and increasing the K/Na ratio [39].

### Photosynthetic pigments

The interaction effect of salinity stress and application of potassium silicate had a significant effect on chlorophyll a, b, total, and carotenoid pigments at the probability level of 1%. Mean comparison of data showed that the content of chlorophyll a, b, and total pigments decreased significantly with increasing salinity level (Additional file 2), but spraying with potassium silicate had a significant effect on chlorophyll a content, especially at a salinity level of 240 mM, increasing the content of chlorophyll b at the levels of 80 and 160 mM and improving the total chlorophyll content at all levels of salinity stress (Table 2). Studies show that with increasing salinity levels due to the accumulation of sodium ions and the reduction of some nutrients such as magnesium, and the reduction of chlorophyll pigments is observed [40]. Other studies also show that the reduction of chlorophyll is caused by the accumulation of stress hormones such as ABA and ethylene under stress conditions and the increase in the activity of the enzyme that breaks down chlorophyll, i.e. chlorophyllase [41]. Foliar spraying with potassium silicate leads to an increase in chlorophyll pigments by reducing ion leakage from the membrane and improving membrane stability [42]. The mean comparison of data showed that the carotenoid content increased under the condition of 80 mM salinity stress, but the increase in salinity levels led to a decrease in the carotenoid content. The use of potassium silicate at all levels led to an increase in carotenoid content under salt stress conditions (Table 2). The increase in carotenoid content can be considered as one of the reasons for improving plant tolerance to salinity [43] and foliar spraying with potassium silicate probably works by increasing carotenoids in the light protection of the photosynthetic system and thus improves photosynthesis [44].

**Table 2:** Effect of different salinity stress with foliar silicate potassium application on some studied traits of *Cichorium intybus* L. plants

Salinity stress (mM)	Silicate potassium	Chl a (mg/g)	Chl b (mg/g)	Total Chl (mg/g)	Carotenoid (mg/g)	MDA content (nmol/g)
0	Control	0.39^g^ ± 0.02	0.34^d^ ± 0.02	0.72^e^ ± 0.04	0.20^cde^ ± 0.1	0.14^i^ ± 0.02
	1	0.44^de^ ± 0.03	0.40^c^ ± 0.02	0.84^c^ ± 0.04	0.24^abc^ ± 0.07	0.11^i^ ± 0.04
	2	0.51^c^ ± 0.03	0.46^b^ ± 0.03	0.96^b^ ± 0.05	0.24^abc^ ± 0.06	0.08^i^ ± 0.02
	3	0.61^a^ ± 0.02	0.56^a^ ± 0.03	1.16^a^ ± 0.06	0.24^abc^ ± 0.06	0.07^i^ ± 0.03
80	Control	0.23^j^ ± 0.01	0.24^e^ ± 0.01	0.48^g^ ± 0.03	0.23^bcd^ ± 0.11	0.74^d^ ± 0.05
	1	0.42^f^ ± 0.02	0.40^c^ ± 0.03	0.82^cd^ ± 0.05	0.24^abc^ ± 0.07	0.36^gh^ ± 0.01
	2	0.45^d^ ± 0.02	0.41^c^ ± 0.03	0.85^c^ ± 0.05	0.24^abc^ ± 0.06	0.30^h^ ± 0.02
	3	0.59^b^ ± 0.03	0.55^a^ ± 0.03	1.14^a^ ± 0.06	0.23^bcd^ ± 0.07	0.18^i^ ± 0.04
160	Control	0.17^l^ ± 0.01	0.06^f^ ± 0.01	0.23^i^ ± 0.01	0.14^fg^ ± 0.06	1.48^b^ ± 0.09
	1	0.19^k^ ± 0.01	0.24^e^ ± 0.02	0.43^h^ ± 0.03	0.29^a^ ± 0.07	0.62^e^ ± 0.07
	2	0.31^i^ ± 0.02	0.22^e^ ± 0.02	0.53^f^ ± 0.03	0.22^bcd^ ± 0.06	0.53^ef^ ± 0.07
	3	0.37^h^ ± 0.03	0.41^c^ ± 0.03	0.78^d^ ± 0.05	0.26^ab^ ± 0.09	0.46^fg^ ± 0.07
240	Control	0.13^m^ ± 0.01	0.03^g^ ± 0.01	0.16^j^ ± 0.01	0.12^g^ ± 0.05	2.27^a^ ± 0.1
	1	0.22^j^ ± 0.02	0.24^e^ ± 0.01	0.47^gh^ ± 0.02	0.27^ab^ ± 0.08	0.94^c^ ± 0.04
	2	0.43^ef^ ± 0.03	0.25^e^ ± 0.02	0.68^e^ ± 0.03	0.18^def^ ± 0.06	0.77^d^ ± 0.07
	3	0.59^b^ ± 0.03	0.25^e^ ± 0.02	0.84^c^ ± 0.03	0.15^efg^ ± 0.05	0.62^e^ ± 0.08

^a,b,c,d,e,f,g,h,i,j,k,l^Means followed by the same letter(s) in each column are not significantly different based on Duncan’s Multiple Range Test (*n* = 3)

### MDA contents

Salinity stress and potassium silicate both had a significant effect on MDA content at the probability level of 1% (Additional file 2). The mean comparison of data shows that MDA content increased strongly with increasing salinity stress level, especially at 240 mM. Foliar spraying with potassium silicate had a significant decrease in MDA content at all salinity levels (Table 2). Similar to our findings, Vafadar et al. [45] reported that salinity induced oxidative stress in *Dracocephalum kotschyi* plants, as evidenced by the increase in electrolyte leakage level and H_2_O_2_ content. Also, studies show that salt stress causes secondary oxidative stress in plants and increases membrane lipid peroxidation, and the use of silicon probably leads to a decrease in ROS production and a rise in the activity of antioxidant enzymes in salt stress [46]. In another study, it was found that the application of silicon leads to tolerance to salinity by improving the antioxidant system and reducing oxidative stress [47]. The salinity phenomenon adversely affects the function of the plasma membrane of plant cells, which leads to membrane lipid peroxidation and MDA production. Membrane damage is associated with ROS production or direct breakdown of polyunsaturated fatty acids [48].

### Total phenol contents

The interaction impact of salinity stress and potassium silicate positively affected total phenol content (Additional file 3). Salinity stress up to the level of 160 mM increased the total phenol content, but the salinity stress at the level of 240 mM decreased the total phenol content. In addition, the use of potassium silicate increased the content of total phenol at all stress levels (Table 3). The results show that potassium silicate can play a positive role in the plant defense system by increasing total phenol levels. The following study examined the concentration of compounds like phenols, flavonoids, and anthocyanins. Based on the findings, salt stress and K_2_SiO_3_ raised secondary metabolites, including flavonoids and phenolic acids. Phenyl compounds such as phenolic acids and flavonoids (formed from the phenylpropanoid metabolic pathway) are important instances of phytochemicals. In addition to acting as phytoalexin or phytoanticipin against biological stress, these substances also have a vital role in plant defense mechanisms under salinity stress conditions as non-enzymatic antioxidant compounds [49]. In addition, this emphasizes the higher total phenol concentration in plants sprayed with K_2_SiO_3_. According to the study of Yaghubi and his colleagues [50], Si obtained from K_2_SiO_3_ can increase the synthesis and accumulation of phenols. Therefore, the higher antioxidant capacity in the plants fed with K_2_SiO_3_ can be associated with the higher concentration of the compounds compared with the control plants. Feeding plants with K_2_SiO_3_ changes the expression pattern of numerous genes, especially those that encode enzymes in the phenylpropanoid metabolic pathway [51].

**Table 3:** Effect of different salinity stress with foliar silicate potassium application on some studied traits of *Cichorium intybus* L. plants

Salinity stress (mM)	Silicate potassium	Total Phenol content (mg gallic acid /gDW)	Total Flavonoid content (mg QE/g DW)	Total anthocyanin content	Inulin (%)
0	Control	46.55^j^ ± 1.31	25.65^j^ ± 0.87	16.01^g^ ± 0.57	39.73^h^ ± 1.42
	1	49.80^i^ ± 1.43	27.41^gh^ ± 0.93	16.93^f^ ± 0.61	40.77^g^ ± 1.45
	2	50.88^h^ ± 1.47	27.85^g^ ± 0.95	17.45^e^ ± 0.62	40.87^g^ ± 1.46
	3	53.34^ef^ ± 1.42	28.95^f^ ± 0.90	18.02^c^ ± 0.59	41.92^f^ ± 1.37
80	Control	51.99^g^ ± 1.13	26.51^i^ ± 0.67	17.63^de^ ± 0.47	39.31^h^ ± 1.05
	1	56.92^d^ ± 1.26	30.18^e^ ± 0.77	18.62^b^ ± 0.50	46.41^e^ ± 1.24
	2	59.45^b^ ± 1.17	31.83^c^ ± 0.72	18.57^b^ ± 0.44	51.64^c^ ± 1.22
	3	64.42^a^ ± 1.29	32.88^b^ ± 0.74	19.26^a^ ± 0.45	52.30^bc^ ± 1.23
160	Control	52.54^fg^ ± 1.26	27.30^gh^ ± 0.77	16.79^f^ ± 0.50	36.56^i^ ± 1.09
	1	58.02^c^ ± 1.43	31.23^d^ ± 0.89	17.87^cd^ ± 0.53	48.99^d^ ± 1.45
	2	59.53^b^ ± 1.78	31.60^cd^ ± 1.08	18.38^b^ ± 0.66	53.01^b^ ± 1.85
	3	64.94^a^ ± 1.97	33.54^a^ ± 1.15	18.45^b^ ± 0.62	56.69^a^ ± 2.02
240	Control	38.05^k^ ± 1.27	16.90^k^ ± 0.7	14.56^h^ ± 0.65	17.01^l^ ± 0.77
	1	51.93^g^ ± 1.89	26.98^hi^ ± 1.15	16.90^f^ ± 0.76	30.94^k^ ± 1.39
	2	52.46^fg^ ± 1.92	27.24^h^ ± 1.16	17.39^e^ ± 0.74	31.91^j^ ± 1.43
	3	54.06^e^ ± 1.99	27.37^gh^ ± 1.17	17.41^e^ ± 0.75	32.09^j^ ± 1.44

^a,b,c,d,e,f,g,h,i,j,k,l^Means followed by the same letter(s) in each column are not significantly different based on Duncan’s Multiple Range Test (*n* = 3)

### Flavonoids contents

Salinity stress, along with potassium silicate, positively affects flavonoid content (Additional file 3). Based on the mean comparison of data, the flavonoid content at 80 and 160 mM salinity levels increased compared to the control, but the 240 mM salinity level decreased the flavonoid content. Application of potassium silicate, especially at salinity levels of 80 and 160 mM, caused a significant increase in flavonoid content (Table 3). Studies also indicate a rise in the expression of flavonoid biosynthetic genes in salt stress conditions [52]. It has been reported that the silicon treatment led to an increase in the activity of phenylalanine ammonia-lyase (PAL), and finally affected the synthesis of phenolic compounds [53].

### Anthocyanin content

The interaction impact of salinit[y and potassium silicate significantly affected anthocyanin content (Additional file 3). Salinity stress at levels of 80 and 160 mM (especially 80 mM) increased the content of anthocyanin, but increasing the level of salinity up to 240 mM increased and decreased the content of this trait. The use of potassium silicate also increased the content of this trait, especially at the salinity level of 80 and 160 mM (Table 3). The results show that silicon could play an effective role in the plant's defense system by improving the anthocyanin content.

#### Inulin content

**Fig. 1:**
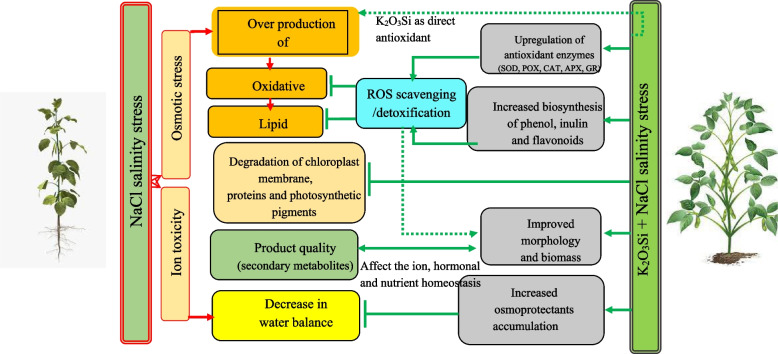
A schematic model for the K_2_O_3_Si-induced mitigation of salinity adverse effects in chicory plants

Salinity stress and potassium silicate significantly affect inulin content (Additional file 3). According to the mean comparison of data, salinity stress at all levels, especially 240 mM, decreased the content of inulin. Foliar spraying with potassium silicate, especially at the level of 1.4 and 1.2 L per thousand liters, improved the content of inulin at all levels of salinity stress (Table 3). Studies show that in conditions of no stress, more inulin content is produced, and stress increases the activity of sucrose phosphate synthetase in the leaf, therefore inulin decreases in stress conditions [54]. A schematic model for the K_2_O_3_Si-induced mitigation of salinity-negative effects in chicory plants is given in Fig. 1.

## Conclusions

The findings suggest that applying K_2_O_3_Si to chicory plants via foliar spray under salinity stress can be an effective strategy to enhance both growth and quality. The study revealed that exposure to NaCl salinity stress reduced root and shoot biomass, leaf chlorophyll content, and inulin accumulation. However, foliar application of K_2_O_3_Si at all salt stress levels improved these parameters. Additionally, K_2_O_3_Si foliar application increased the plants’ tolerance to salinity stress by reducing MDA levels and increasing phenolic compounds and potassium content. Nevertheless, further research is required to understand the molecular-level regulatory mechanisms of K_2_O_3_Si treatment in mitigating salinity stress in chicory.

### Supplementary Information

**Supplementary Material 1. **

**Supplementary Material 2. **

**Supplementary Material 3. **

## Data Availability

No datasets were generated or analysed during the current study.
