# Supplementary material for: Foliar-applied silicate potassium modulates growth, phytochemical, and physiological traits in Cichorium intybus L. under salinity stress
Source: BMC Plant Biol. 2024 Apr 16;24:288. doi: 10.1186/s12870-024-05015-6 (PMC11020321; doi:10.1186/s12870-024-05015-6)
Supplement: Supplementary file 1 — Supplementary Material 1. [file 12870_2024_5015_MOESM1_ESM.docx]

**Additional file 1** Analysis of variance (ANOVA) for the studied traits in *Cichorium intybus* L. plants under different salinity stress (Factor a) with silicate potassium sprayed (Factor b).

| **Source** | **df** | **Mean Square** |  |  |  |  |  |  |
| --- | --- | --- | --- | --- | --- | --- | --- | --- |
|  |  | **Shoot Dry Weight** | **Root Dry weight** | **Root-toshoot ratio** | **Fv/Fm** | **Na+ content** | **K^+^ content** | **K/Na** |
| Block | 2 | 9.24 ^*^ | 0.83 ^*^ | 1.15 ^*^ | 0.0007 ^ns^ | 0.10 ^*^ | 30.21 ^*^ | 4.64 ^*^ |
| a | 3 | 124.05^**^ | 4.76^**^ | 162.49^**^ | 0.081** | 4.34^**^ | 41.02^**^ | 36429.49^**^ |
| b | 3 | 39.90^**^ | 1.81^**^ | 35.25^**^ | 0.013^**^ | 0.37^**^ | 42.40^**^ | 297.85^**^ |
| a ×b | 9 | 2.03^**^ | 0.13^**^ | 28.13^**^ | 0.001** | 0.07^**^ | 3.93^**^ | 20.94** |
| Error | 30 | 0.14 | 0.006 | 0.05 | 0.0002 | 0.002 | 0.15 | 0.86 |

Note: *: Significant at the 0.05 probability level; **: Significant at the 0.01 probability level; ns: Not significant.
