# Supplementary material for: Foliar-applied silicate potassium modulates growth, phytochemical, and physiological traits in Cichorium intybus L. under salinity stress
Source: BMC Plant Biol. 2024 Apr 16;24:288. doi: 10.1186/s12870-024-05015-6 (PMC11020321; doi:10.1186/s12870-024-05015-6)
Supplement: Supplementary file 2 — Supplementary Material 2. [file 12870_2024_5015_MOESM2_ESM.docx]

**Additional file 2** Analysis of variance (ANOVA) for the studied traits in *Cichorium intybus* L. plants under different salinity stress (Factor a) with silicate potassium sprayed (Factor b).

**MDA**

**Mean Square**

**df**

**Source**

**Carotenoid**

**Total Chl**

0.250

0.06

**Chl a Chl b**

**content**

Block 2 0.01 ^*^ 0.01 ^*^ ^* *^ 0.10 ^*^ a 3 0.11^**^ 0.17^**^ 0.53^**^ 0.007^**^ 2.50^**^ b 3 0.21^**^ 0.15^**^ 0.70^**^ 0.015^**^ 1.69^**^ a ×b 9 0.01^**^ 0.005^**^ 0.01^**^ 0.004^**^ 0.29^**^ Error 30 0.0001 0.0003 0.0007 0.0009 0.004

Note: *: Significant at the 0.05 probability level; **: Significant at the 0.01 probability level; ns:

Not significant.
