# Supplementary material for: Foliar-applied silicate potassium modulates growth, phytochemical, and physiological traits in Cichorium intybus L. under salinity stress
Source: BMC Plant Biol. 2024 Apr 16;24:288. doi: 10.1186/s12870-024-05015-6 (PMC11020321; doi:10.1186/s12870-024-05015-6)
Supplement: Supplementary file 3 — Supplementary Material 3. [file 12870_2024_5015_MOESM3_ESM.docx]

**Additional file 3** Analysis of variance (ANOVA) for the studied traits in *Cichorium intybus* L. plants under different salinity stress (Factor a) with silicate potassium sprayed (Factor b).

| **Source** | **df** | **Mean Square** |  |  |  |
| --- | --- | --- | --- | --- | --- |
|  |  | **Phenol** | **Flavonoid** | **Anthocyanin** | **Inulin** |
| Block | 2 | 107.69^*^ | 39.99^**^ | 17.15^**^ | 89.91^**^ |
| a | 3 | 315.52^**^ | 100.98^**^ | 8.84^**^ | 1085.04^**^ |
| b | 3 | 298.38^**^ | 102.41^**^ | 9.59^**^ | 382.90^**^ |
| a ×b | 9 | 16.64^**^ | 8.90^**^ | 0.42^**^ | 39.67^**^ |
| Error | 30 | 0.28 | 0.10 | 0.04 | 0.28 |

Note: *: Significant at the 0.05 probability level; **: Significant at the 0.01 probability level; ns:

Not significant.
